# Novel SPEG variant cause centronuclear myopathy in China

**DOI:** 10.1002/jcla.23054

**Published:** 2019-10-18

**Authors:** Jia Tang, Wei Ma, Yangran Chen, Runze Jiang, Qinlong Zeng, Jieliang Tan, Hongqing Jiang, Qing Li, Victor W. Zhang, Jing Wang, Hui Tang, Liangping Luo

**Affiliations:** ^1^ Department of Medical Imaging Center The First Affiliated Hospital of Jinan University Jinan University Guangzhou China; ^2^ Medical Genetics Center Jiangmen Maternity and Child health Care Hospital Jiangmen China; ^3^ Department of Biology School of Basic Medicine Jiamusi University Jiamusi China; ^4^ AmCare Genomics Laboratory International BioIsland Guangzhou China

**Keywords:** centronuclear myopathy, medical exome sequencing, new clinical symptoms, novel variant, SPEG

## Abstract

**Background:**

Centronuclear myopathy (CNM), a subtype of congenital myopathy (CM), is a group of clinical and genetically heterogeneous muscle disorders. Centronuclear myopathy is a kind of disease difficult to diagnose due to its genetic diversity. Since the discovery of the SPEG gene and disease‐causing variants, only a few additional patients have been reported.

**Methods:**

A radiograph test, ultrasonic test, and biochemical tests were applied to clinical diagnosis of CNM. We performed trio medical exome sequencing of the family and conservation analysis to identify variants.

**Results:**

We report a pair of severe CNM twins with the same novel homozygous SPEG variant c. 8710A>G (p.Thr2904Ala) identified by clinical trio medical exome sequencing of the family and conservation analysis. The twins showed clinical symptoms of facial weakness, hypotonia, arthrogryposis, strephenopodia, patent ductus arteriosus, and pulmonary arterial hypertension.

**Conclusions:**

Our report expands the clinical and molecular repertoire of CNM and enriches the variant spectrum of the SPEG gene in the Chinese population and helps us further understand the pathogenesis of CNM.

## 1 INTRODUCTION

Centronuclear myopathy (CNM) is a subgroup of congenital myopathy,^1^ characterized by skeletal muscle weakness and atrophy.^2^ The phenotypes of CNM are commonly present at newborn, infant, and early childhood.

The clinical spectrum of CNM is diverse among affected individuals, and the genetic etiology is heterogeneous. Only about 60%‐80% of CNM can be explained by mutations in DNM2, RYR1, CACNA1S, BIN1, and MTM1 genes.^3^, ^4^, ^5^, ^6^, ^7^, ^8^ The rest have a genetic basis of the remaining CNMs remain unidentified.

SPEG is a protein highly expressed in striated muscle cells and cardiomyocytes, which play an important role in the differentiation of vascular smooth muscle cell in early life.^9^, ^10^, ^11^, ^12^ Recessive SPEG (Striated muscle enriched protein kinase) mutations were first identified in CNM patients by Agrawal in 2014.^13^ To date, SPEG mutations have been identified in eight CNM and 1 CM patients.^13^, ^14^, ^15^, ^16^, ^17^ Most of these patients had severe hypotonia and muscle weakness.

Here, we report an additional pair of fraternal CNM twins with a novel homozygous SPEG variant, c.8710A>G (p.Thr2904Ala). We have also reviewed clinical findings of reported 11 CNM patients and discussed the genotype‐phenotype correlations, to further our understanding of SPEG‐related CM.

## 2 MATERIALS AND METHODS

### 2.1 Specimens and DNA preparation

Blood samples were collected and prepared at Jiangmen Maternity and Child Health Care Hospital. Approval of the Jiangmen Maternity and Child Health Care Hospital ethics committee was obtained, as well as informed consent from all adult participants and parents of the participating children. Genomic DNA was extracted from peripheral blood using the Solpure Blood DNA kit (Magen) according to the manufacturer's instructions.

### 2.2 Target capture and sequencing

The isolated genomic DNA was fragmented by the Q800R Sonicator (Qsonica) to generate 300‐500 bp DNA fragments for library preparation and paired‐end sequencing according to manufacturer's Illumina library preparation protocols. Custom designed NimbleGen SeqCap probes (Roche NimbleGen) targeting about 4000 genes with known clinical relevance were used for in‐solution hybridization to enrich target gene sequences. Enriched DNA samples were indexed and sequenced on NextSeq500 sequencer (Illumina) with 100‐150 cycles of paired‐end reads, according to the manufacturer's protocols. Sanger sequencing was used to confirm the variants identified by NGS and determine the segregation of the variant in the family. Sanger sequencing was performed as previously described.^18^

### 2.3 Variant annotation and interpretation

The raw reads were filtered to generate “clean reads” by removing adapters and low‐quality reads (Q20). Clean reads were mapped to the reference human genome hg19 (2009‐02 release) with BWA 0.7.15. Unmapped reads were removed. SNVs and short indels were scored and reviewed by using NextGENe 2.4.1.2 and GATK 3.5. In addition, the eCNVscan was used to detect large exonic deletions and duplications.^19^ The normalized coverage depth of each exon of a test sample was compared with the mean coverage of the same exon in the reference file, to detect copy number variants (CNVs).^20^ All short variants were annotated with databases including 1000 Genomes, dbSNP (build 148), gnomAD (http://gnomad.broadinstitute.org/), ClinVar, HGMD, and OMIM. Common variants (frequency >1% in database) were discarded. Rare or novel variant sites at which genotype of patient are different with parents were further investigated for its conservation of amino acid change, and structural/functional regions of the protein to evaluate potential pathogenicity of the variant. Mutation Taster, http://www.mutationtaster.org/. Variant's interpretation was performed according to the American College of Medical Genetics (ACMG) guidelines.^21^ Conservation of amino acids around the variant sites was analyzed with CLUSTAL among 10 species (human [Homo sapiens], brown rats [Rattus norvegicus], domestic cattle [Bos Taurus], chimpanzees [Pan troglodytes], macaques [Macaca mulatta], mouse [Mus musculus], junglefowl [Gallus gallus], clawed frog [Xenopus], zebrafish [Danio rerio], and leaf kiss chimera [Callorhinchus milii]). We used GPS (http://gps.biocuckoo.cn/online.php) to predict kinase‐specific phosphorylation sites in SPEG.

### 2.4 Minigene molecular cloning, transfection, and RT‐PCR

Genomic DNA was extracted from whole‐blood samples. Mutagenesis was carried out according to the PCR mutagenesis protocol Site‐directed mutagenesis. Wild‐type (wt) minigene SPEG exon 36 with intronic was used as template to generate variants. We subjected 36 exons of SPEG with the intronic boundaries to PCR‐Sanger sequencing. PCR was performed under the following conditions: initial denaturation at 95°C for 3 minutes, followed by 35 cycles of 95°C for 30 seconds, 62°C for 30 seconds, and 72°C for 30 seconds.

The identified variant of c. 8710A>G is located in the region of exon 36. To study the effect of this variant on the splicing pattern, DNA fragment of 793 bp encompassing exon 36 and the flanking intronic sequences of SPEG was amplified from the genomic DNA. The primers (Minigene‐F/R) used for the generation of the minigene constructs are listed in Table S1. Each PCR product was digested with the BglII and MluI restriction enzymes and cloned into the pCAS2 vector, which had also been digested with BamHI and MluI. All of the selected clones were sequenced, and the verified clones, referred to as the wild‐type (pSPEG‐c.8710A) and mutant (pSPEG‐c.8710G) clones, were retained for expression experiments.

293T cells were grown in 5% CO2 incubator at 37°C in Dulbecco's modified Eagle's medium supplemented with 10% fetal bovine serum (Bovogen). Transfection of 293T cells with pSPEG‐c.8710A, pSPEG‐c.8710G, and the empty pCAS2 vector using Lipofectamine 3000(from Invitrogen). Twenty four hours after transfection, cells were collected and total RNA was extracted using TRIzol (Invitrogen). Then, 1.5μg of total RNA was reverse transcribed using a Reverse Transcription System according to the manufacturer's instructions (Invitrogen). Following RNA retrotranscription, 200 ng of complementary DNA from the three constructs mentioned above was PCR amplified using the primers (reverse transcriptase–PCR‐F/R) shown in Table S1. The PCR products were then separated on a 1% agarose gel, and individual bands were excised and sequenced using specific primers (SEQ primer‐F/R, Table S1).^22^, ^23^

## 3 RESULT

### 3.1 Clinical description

**Figure 1:**
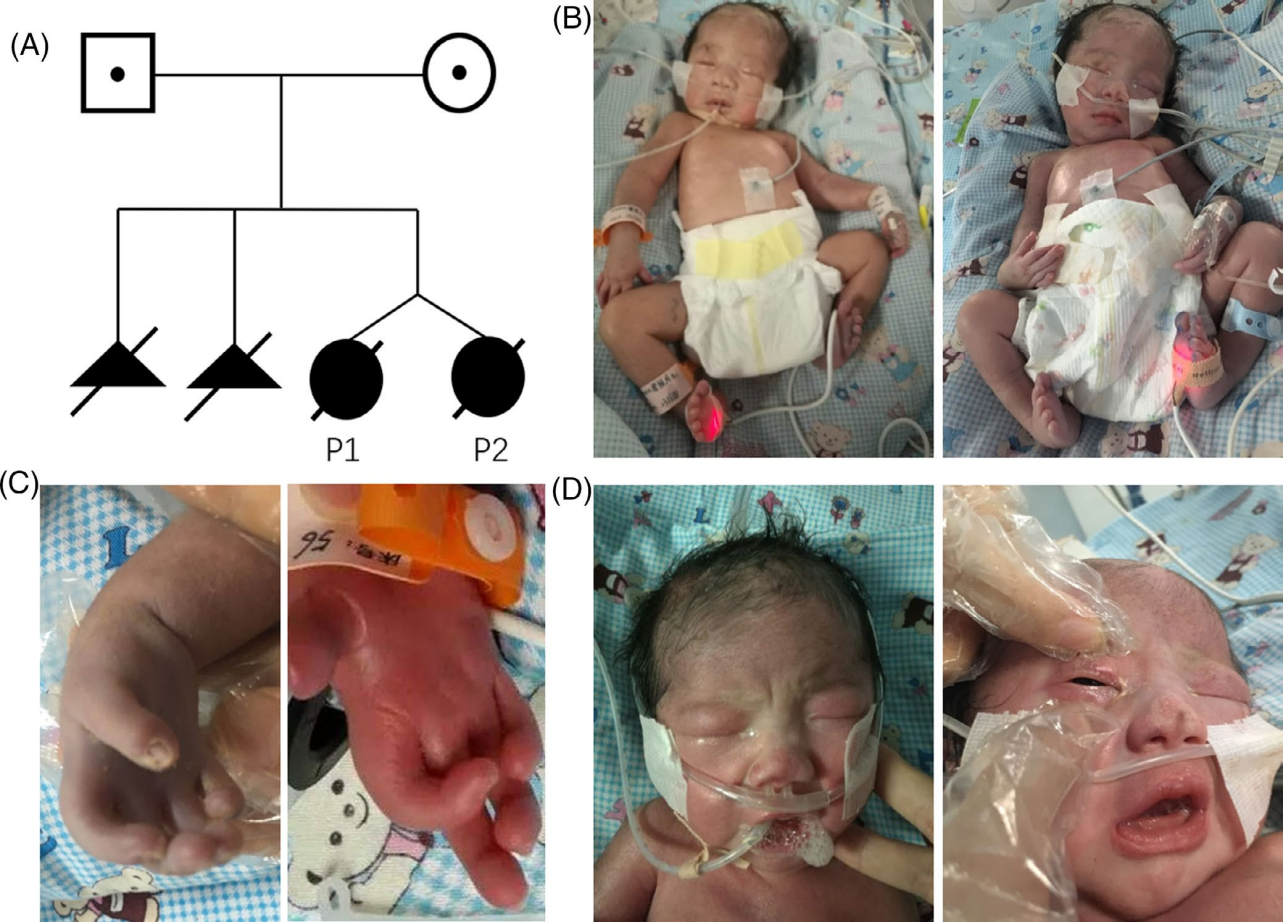
Family pedigree and Phenotypic characteristics of two patients (left:patient1; right:patient2). A, Pedigree of the family under study. B, facial weakness, limb deformities, and strephenopodia. C, abnormal palmprint feature and arthrogryposis. D, ophthalmoplegia

Parents of our twin patients have three pregnancies. The previous two pregnancies resulted in abortion. The third pregnancy resulted in the fraternal twins described here. (Figure 1A). During the first pregnancy in 2011, the fetus was found to have strephenopodia and limb deformities by ultrasound at 24th week, and abortion was performed. During the second pregnancy in 2013, mother reported decreased fetal movement. Ultrasound showed limb deformities and strephenopodia at 25th week of pregnancy, and abortion was performed. Karyotype analysis of the two fetuses showed no chromosomal abnormalities.

During the third pregnancy in 2016, mother reported fetal akinesia. Ultrasound showed twin with strephenopodia and increased echogenicity of the subcutaneouslayer and muscularlayer of the limb. Two babies were delivered at 39th week by cesarean section, weighed 2280 g and 2320 g. Their Apgar scores of both twins were 10‐10‐10. They were presented with facial weakness, drooping eyelids, arthrogryposis, respiratory insufficiency, hypotonia, axial muscle weakness, easily fractured, and strephenopodia (Figure 1B‐D). The two neonates were cared for in an incubator with an oxygen inhalation apparatus.

The Ultrasonic cardiogram of the twin showed patent ductus arteriosus, patent foramen ovale, and pulmonary arterial hypertension. Electrocardiograms showed nodal tachycardia and right atrium abnormality. The chest radiograph showed a diffused fine reticular shadowing and decreased aeration throughout both lungs with the air bronchograms and localized ground‐glass appearance. In addition, confluent reticulogranular shadowing was observed in the right upper lung, together with an opacification in the bilateral hilar zones. Considering the clinical symptoms, the neonatal hyaline membrane disease was diagnosed and the re‐examination after treatment was also advised.

The biochemical values of twin1 and twin2 at 14 hours after birth were respectively: Creatine Kinase (CK) 187 U/L, 207 U/L (25‐200 U/L), creatine kinase isoenzymes (CK‐MB) 39 U/L, 43 U/L (0‐30 U/L), lactate dehydrogenase(LDH) 383 U/L, 342 U/L (114‐240 U/L), HBDH 341 U/L, 307 U/L (72‐182 U/L), Aspartate aminotransferase(AST) 37 U/L, 30.7 U/L (4‐50 U/L), and AST/Alanine aminotransferase (ALT) 6.17, 5.2 (0.5‐1.5). The indices of the biochemical blood tests indicated cardiomyopathy. The twin expired at 1 week of age, after life‐threatening dyskinesia. Parents declined muscle biopsy. Instead, Medical exome sequencing was performed on twin1 and her healthy parents.

### 3.2 Genetic results

**Figure 2:**
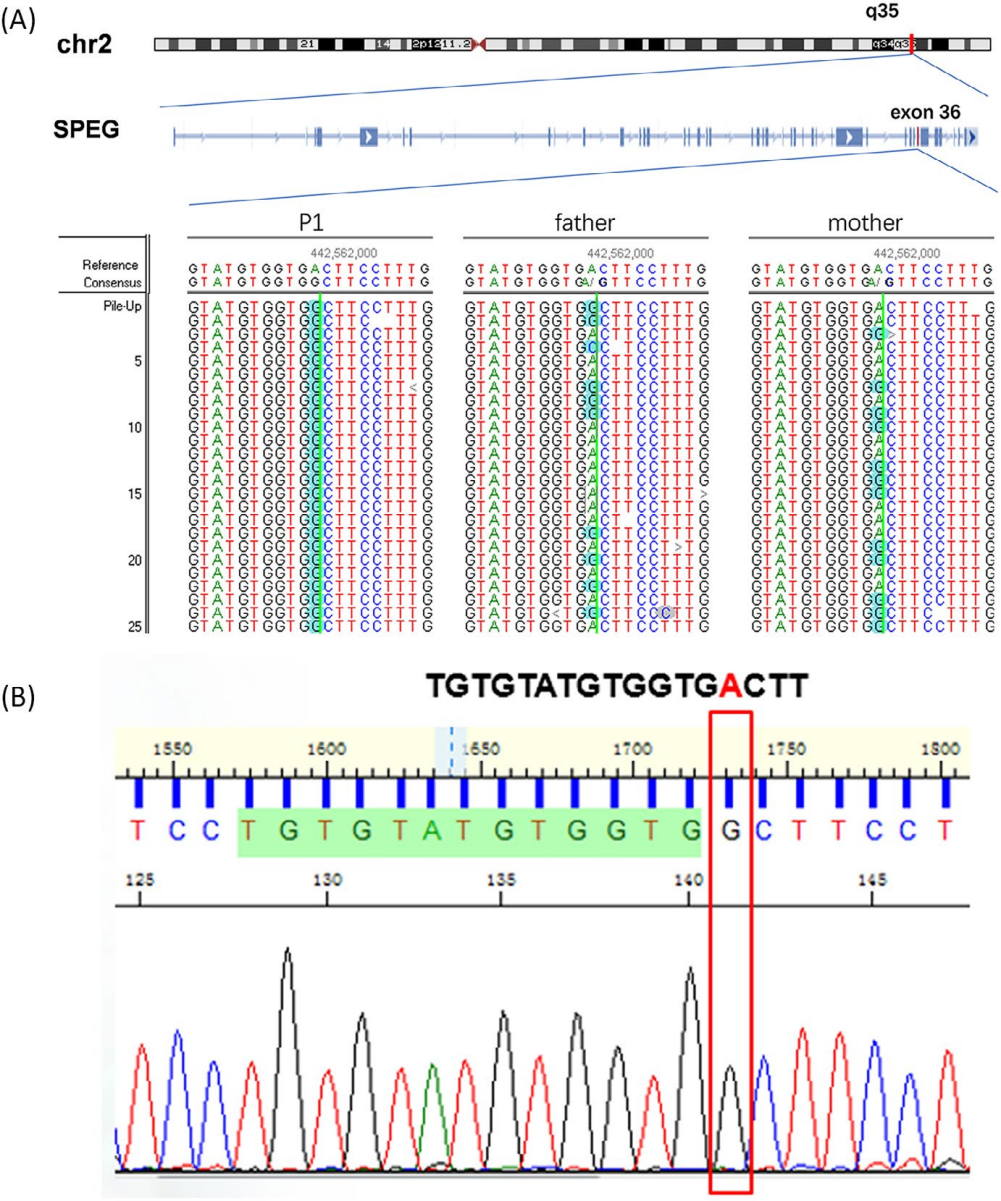
Sequencing of SPEG variant c.8710A>G. A, c.8710A>G variant was detected by medical exome sequencing, Patient1 (left), father (middle), and mother (right). B, Sanger sequencing of Patient2

**Figure 3:**
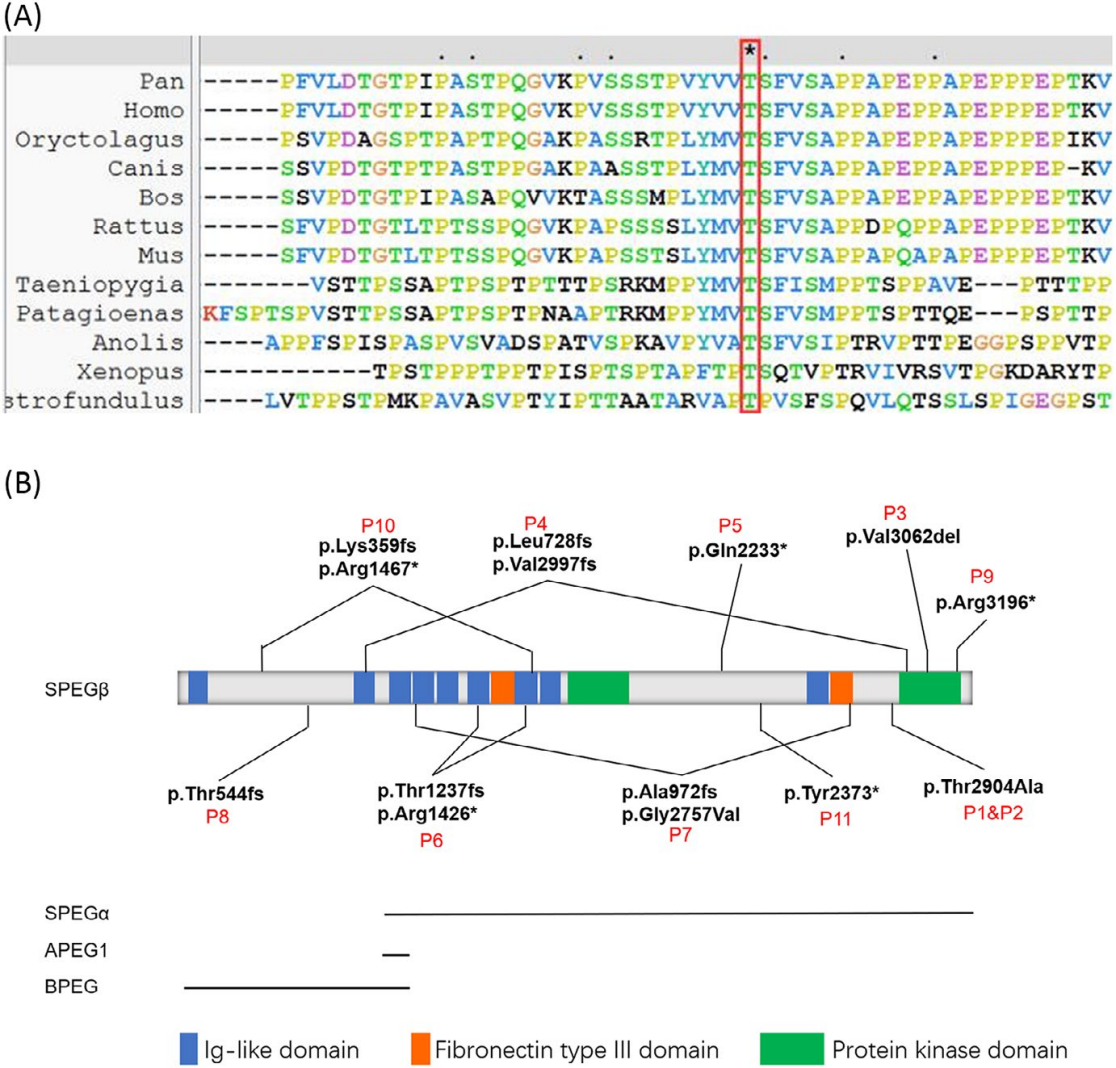
Multiple sequence alignment of the SPEG from 10 different species and reported pathogenic mutations. (A). conservation analysis result of mutation c.8710A>G, p.Thr2904Ala (B). SPEG protein domains and reported pathogenic mutations

Genetic counselling was provided, and informed consent was obtained for genetic analysis. Trio medical exome sequencing identified a homozygous variant of unknown significance in exon 36 of SPEG, c.8710A>G, p.Thr2904Ala (Figure 2A). The variant was heterozygous in both parents (Figure 2A) and homozygous in the affected sister by Sanger sequencing (Figure 2B). The amino acid threonine at position 2904 is highly conserved (Figure 3A). It is near the protein kinase domain, which is critical for SPEG function (Figure 3B).

Our patient's phenotype and family history are highly specific for CNM, which are caused by a variation in the SPEG. The variant, c.8710A>G (rs535105065), has been reported in GnomAD and database shows 14 times in the heterozygous state of the Asian population (minor allele frequency 0.00005688), which is extremely low frequency. c.8710A>G is a kinase‐specific phosphorylation site in SPEG by GPS predicted the result. Polyphen result show variant is probably damaging. Mutation taster result show protein features might be affected, and conservation analysis show variant is highly conserved. We report a pair of fraternal CNM twins with a homozygous SPEG variant, both parents were heterozygous. In summary, c.8710A>G is an uncertain significance variant.

### 3.3 Impact of the c.8710A>G mutation on SPEG mRNA splicing

Mutation taster result of c.8710A>G show may change splice site and effect SPGE mRNA splicing. An in vitro minigene system was used to variant c.8710A>G effect on splicing, and the experiment result showed no effect on splicing.

## 4 DISCUSSION

Here, we report a pair of fraternal CNM twins with new clinical symptoms and an homozygous SPEG variant, c.8710A>G, p.Thr2904Ala. The twins showed patent ductus arteriosus, patent foramen ovale, pulmonary arterial hypertension, and strephenopodia during pregnancy. These new symptoms expand the clinical spectrum of CNM.

So far, there have been 8 CNM and 1 CM patients with SPEG mutations reported. The clinical and molecular findings of all 11 patients reported so far including ours are summarized in Table 1.

**Table 1:** Clinical features and molecular evidence of individuals carrying SPEG mutations

Patient/sex	P1/F	P2/F	P3/M^15^	P4/F^15^	P5/F^13^	P6/F^13^	P7/F^13^	P8/M^16^	P9/M^16^	P10/F^14^	P11/M^16^
Age	died at 3 d	died at 5 d	Died at 17 y	6.5 y	Died at 3 wk	6 y	1.5 y	3 y	7 y	10 y	Died at 19 wk
SPEG exons	Exon 36	Exon 36	Exon 38	Exon 10 and 38	Exon 30	Exons 18 and 13	Exons 10 and 35	Exon 4	Exon 40	Exon 4 and 20	Exon 30
Allele1	c.8710A>G, p.Thr2904Ala	c.8710A>G, p.Thr2904Ala	c.9185_9187delTGG; p.Val3062del	c.2183delT; p.Leu728fs	c.6697C>T; p.Gln2233*	c.4276C>T; p.Arg1426*	c.2915_2916delCCinsA; p.Ala972fs	c.1627‐1628insA; p.Thr544fs	c.9586C>T; p.Arg3196*	c.1071_1074dup; p.Lys359fs	c.7119C>A; p.Tyr2373*
Allele2	same as above	same as above	same as above	c.8962_8963ins25; p.Val2997fs	same as above	c.3709_3715 + 29del36; p.Thr1237fs	c.8270G>T; p.Gly2757Val	same as above	same as above	c.4399C>T; p.Arg1467*	same as above
Family history	Non‐consanguineous but both heterozygous at mutation site	Non‐consanguineous but both heterozygous at mutation site	Consanguineous parents, one healthy sister	No known consanguinity	Consanguineous parents, two sisters died early	No known consanguinity	No known consanguinity, sibling died early	parents from village in Turkey	Likely consanguineous	Non‐consanguineous	Consanguineous parents
Birth history	Full term,poor fetal movements,hypotonic,strephenopodia,Abnormal limbs	Full term,poor fetal movements,hypotonic,strephenopodia,Abnormal limbs	Full term, severely hypotonic	Full‐term, hypotonic	Full‐term, breech delivery, severely hypotonic	Severely hypotonic	Born at 36 wk of gestation, severely hypotonic	Full‐term, hypotonic	Full‐term, poor fetal movements	Uneventful pregnancy, hypotonic	Uneventful pregnancy, severely hypotonic
Neurological findings	NA	NA	symmetric atrophy of lower extremities, wheel chair bound at 17 y	normal early motor milestones, walked at 2 y, unable to run or jump	Died of severe muscle weakness	Sit unsupported at 2.5 y, unable to walk unsupported	Head control at 16 mo, sit unsupported at 18 mo	Head control—6 mo, sit unsupported —12 mo, unable to walk	Head control at 18 mo, sitting at 30 mo, walking—4 y	sit—11 mo, walk—30 mo, short distances	Contracture of right ankle and lacked deep tendon reflex, antigravity movement at 1 wk
Eye findings	Ophthalmoplegia	Ophthalmoplegia	Ophthalmoplegia	Ophthalmoplegia, bilateral ptosis	No known evaluation	Ophthalmoplegia	None	ophthalmoplegia, mild ptosis	None	None	None
Respiratory issues	3 d of NICU stay for respiratory issues	5 d of NICU stay for respiratory issues	non‐invasive ventilation during night, recurrent pneumonia	Weak cough	Insufficient respiratory efforts	Tracheostomy, mechanical ventilation dependent	brief NICU stay for respiratory issues, no assisted ventilation	NICU for apnea, no intubations, recurrent lung infections	non‐invasive ventilation during first 48 h of life	None	Intubation required immediately after birth, weaned at 10 wk for palliative care
Feeding issues	NONE	NONE	Gastrostomy tube from age 6	gastrostomy tube	Gastrostomy tube early in life	Gastrostomy tube early in life	NG feeding	None	NG feeding until day 13	Gastrostomy tube from age 9	Gastrostomy tube
Cardiac issues	Dilated cardiomyopathy	Dilated cardiomyopathy	Dilated cardiomyopathy at age 7, severe mitral valve insufficiency	No cardiomyopathy at 3 y 10 mo, sinus tachycardia	No cardiac evaluation	Dilated cardiomyopathy	Dilated cardiomyopathy, mitral valve insufficiency	None	Dilated cardiomyopathy mild mitral insufficiency	Reduced myocardial mitral valve insufficiency contraction, no ventricular dilation at 5 y	Enlarged atria, abnormal trabeculation of left ventricle
Skeletal issues	Multiple joint inactivity	Multiple joint inactivity, right humeral fracture at birth	Torsion scoliosis	Ulnar fracture at age 4, condyle fracture at age 5, tibia fracture at age 11 (all after trauma)	Not applicable	None	None	Pectus excavatum and mild scoliosis	None	Scoliosis developed at age 4	Not applicable

The interaction with MTM1 was considered the main pathogenic pathway of SPEG function. The region in the C‐terminal (amino acid 2530‐2674) is required for interaction with MTM1. These can explain most genotype‐phenotype correlations of the patients. It was discovered in a mouse that SPEG dysfunction produces a myopathy by affecting Ca^2+^ current function of the voltage sensor, calcium release from the SR and consequently reducing muscle contractility.^24^, ^25^ Recent studies confirmed that the protein kinase domain II is actually the key domain that controls the Ca^2+^ re‐uptake through regulating SERCA2a.^26^ These indicate that dysfunction of protein kinase domain II of SPEG may cause CNM because of unbalanced calcium homeostasis through the SERCA2a pathway. The mutation p.Arg3196*, p.Val3062del, p.Val2997Glyfs*52 also occurred just before the interaction region with MTM1 and may cause dysfunction of protein kinase domain II.

SPEG is alternatively spliced into four tissue‐specific isoforms that were identified in murine models including SPEGα, aortic preferentially expressed gene‐1 (APEG‐1), SPEGβ, and brain preferentially expressed gene (BPEG). SPEG has a critical role in skeletal and cardiac function, and SPEGα and SPEGβ are highly expressed in skeletal and cardiac muscle.^10^ Clinical data from Patients 4, 8, and 10 suggest that SPEGα may partially rescue mutations affecting only SPEGβ, possibly preserving cardiac function. SPEGα and SPEGβ are proteins within the junctional membrane complex (JMCs) that reported to regulatory junctophilin‐2 (JPH2) phosphorylation, which is a key role for transverse tubules maintenance in cardiac myocytes.^16^ Clinical data from patients^3^, ^5^, ^6^, ^7^, ^9^, ^11^ carried mutation affecting SPEGα and SPEGβ. These findings suggest the disease is more severe when both isoforms are affected. Our two patients who carried variant p.Thr2904Ala affecting SPEGα and SPEGβ were severe and died within a week.

In conclusion, we diagnosed a CNM family and pathogenesis with medical exome sequencing guided by extraordinary rare clinical information. Molecular gene diagnosis may contribute to the documentation of molecular heterogeneity and racial differences in CM patients. Differential impact of mutations reported may help to understand the genotype‐phenotype correlations and will provide the basis for new molecular and treatment strategies.
