# Supplementary material for: Novel SPEG variant cause centronuclear myopathy in China
Source: J Clin Lab Anal. 2019 Oct 18;34(2):e23054. doi: 10.1002/jcla.23054 (PMC7031609; doi:10.1002/jcla.23054)
Supplement: Supplementary file 1 [file JCLA-34-e23054-s001.doc]

Supplementary Table 1

| The primers used for generation of the minigene constructs | Minigene-F1 | GCCGGATCCCAAGGCACCACGGTGATGATTT |
| --- | --- | --- |
|  | Minigene-R1 | AATACGCGTTCTGGCACATTTGAACGCAATC |
| The primer used for generation mutation | Mutation-F2 | CCTGTGTATGTGGTGGCTTCCTTTGTGTCTGCACC |
|  | Mutation-R2 | GGTGCAGACACAAAGGAAGCCACCACATACACAGG |
| The primer used for verification of the minigene with PCR and sequencing | F3 | CAAGGCACCACGGTGATGATTT |
|  | R3 | TCTGGCACATTTGAACGCAATC |
| The primers used for reverse tranacription of the RNA extracted from minigene constructs | RTpcr-F4 | CTGACCCTGCTGACCCTCCT |
|  | RTpcr-R4 | TTGCTGAGAAGGCGTGGTAGAG |
| The primers used for sequencing the reverse transcribed cDNAs from minigene constructs | SEQ-F5 | GTGAAGACAGGGAAGAATGGTAG |
|  | SEQ-R5 | ACTGAGAAACCACGCATTAGC |
